# Whole genome sequencing in clinical practice

**DOI:** 10.1186/s12920-024-01795-w

**Published:** 2024-01-29

**Authors:** Frederik Otzen Bagger, Line Borgwardt, Andreas Sand Jespersen, Anna Reimer Hansen, Birgitte Bertelsen, Miyako Kodama, Finn Cilius Nielsen

**Affiliations:** Center for Genomic Medicine, Rigshospitalet, University of Copenhagen, Copenhagen, Denmark

**Keywords:** Whole genome sequencing, Clinical bioinformatics infrastructure, Variant filtering and interpretation, Functional variant testing

## Abstract

Whole genome sequencing (WGS) is becoming the preferred method for molecular genetic diagnosis of rare and unknown diseases and for identification of actionable cancer drivers. Compared to other molecular genetic methods, WGS captures most genomic variation and eliminates the need for sequential genetic testing. Whereas, the laboratory requirements are similar to conventional molecular genetics, the amount of data is large and WGS requires a comprehensive computational and storage infrastructure in order to facilitate data processing within a clinically relevant timeframe. The output of a single WGS analyses is roughly 5 MIO variants and data interpretation involves specialized staff collaborating with the clinical specialists in order to provide standard of care reports. Although the field is continuously refining the standards for variant classification, there are still unresolved issues associated with the clinical application. The review provides an overview of WGS in clinical practice - describing the technology and current applications as well as challenges connected with data processing, interpretation and clinical reporting.

**Supplementary Information:**

The online version contains supplementary material available at 10.1186/s12920-024-01795-w.

## Background

The human genome project was a ground-breaking scientific endeavour that not only gave us a near complete map of our genetic code but also paved the way for new innovative sequencing technologies and computational methods that have enabled the clinical application of genomics [1–4]. While DNA sequencing dates back to the late 1970s [5], it was not until the beginning of the 90s that sequencing, with advent of semi-automized four-color dye sequencing [6], became available for routine clinical use. Since then, the development of Next Generation Sequencing (NGS), has revolutionized the field, enabling the analysis of entire genomes in a fast and cost-effective manner [7, 8]. At this stage the last hard-to-sequence bits of the human genome have been mapped, and hundreds of thousands of people have had their entire genome sequenced [9].

The capacity of NGS has steadily increased and with the latest generation of sequencing platforms, an entire human genome can be sequenced within 2 days at the price of a few hundred dollars. The relatively modest costs per analysis, combined with excellent data quality [10], make whole genome sequencing (WGS) a valuable source of information in many clinical situations. Compared to other genomic analysis, archived WGS data moreover have the potential to serve as a lifelong companion for patients that can be reanalysed and reinterpreted several times along the patient journey.

Similar to other medical developments, the clinical implementation of WGS requires that we closely consider advantages compared to the current practice, as well as the limitations and ethical issues of the technology. In this review, we describe the elements and concerns of WGS in clinical practice. Following the trail of the patient sample, we explain the technological platforms and the data infrastructure as well as the processing and interpretation of the results. Finally, we outline and discuss the clinical applications, guidelines and clinical reporting.

## Whole genome sequencing

**Fig. 1:**
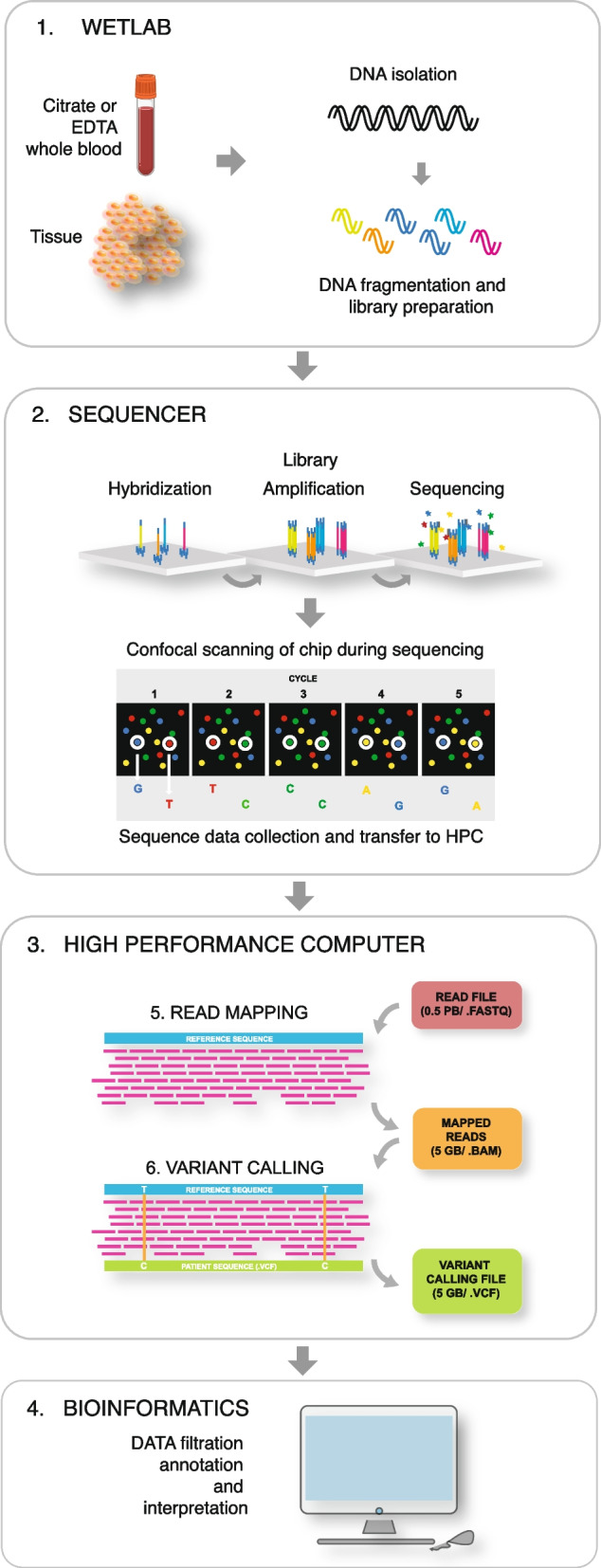
Schematic representation of the WGS laboratory and bioinformatics flow. Short-read WGS protocols can in general be divided into four separate steps: 1. Sample preparation, 2. Library preparation, 3. Cluster generation, and 4. Sequencing. Panel 1, WGS is routinely performed with DNA from EDTA or citrate stabilized whole blood or surgically removed or biopsy tissue. DNA is isolated by conventional methods, but to facilitate CNV detection high molecular DNA is preferred. Historically, WGS required a DNA amplification step, but with newer protocols this step is no longer needed. Omission of the amplification step eliminates the PCR-bias and provides a more uniform coverage and quality [12]. The library is generated by fragmenting the high molecular DNA followed by ligation of adapters that will bind to the linker DNA on the chip surface. Moreover, barcodes allowing pooling of samples from different patients on the same chip may be attached. Panel 2, The libraries are subsequently loaded onto a flow cell and placed on the sequencer, after which the individual DNA fragments are clonally amplified by a polymerase, generating small single-stranded clusters of the particular fragments. The sequencing is in principle a conventional Sanger sequencing [5], where elongation is initiated by the addition of a sequence primer and polymerase and the nucleotide sequence is determined by the incorporation of complementary fluorescent-tagged nucleotide terminators. The fluorescent signal from the incorporated terminators is detected by scanning the chip and the individual clusters with a high-resolution confocal fluorescence laser detector after every round of nucleotide incorporation. Panel 3, Data are compiled in a fastq file that is being transferred to the high performance computer (HPC). In the HPC the reads are mapped and compiled in a .BAM file before variants are called listed in a .VCF file. Panel 4, The VCF is finally uploaded to the interpreters in the genomic laboratory for filtration, annotation and prioritization

NGS was originally referred to as massive parallel sequencing (MPS) [11] describing the parallel processing and sequencing of millions of DNA fragments in small vesicles or on a solid phase and the subsequent alignment of the sequence reads to a reference genome. The output of NGS has steadily increased since 2005 [8], where it was suitable for sequencing of smaller selected parts of the genome, to WGS that became possible around 2010 and was FDA approved in 2018. The laboratory procedures are relatively simple and can be performed in any conventional molecular biology laboratory. The general WGS workflow is outlined in Fig. 1.

The major difference between WGS and other types of NGS analyses is basically that there is no sequence capture and the amount of data generated. Until a few years ago, the cost of WGS was relatively high, but with the advent of second-generation chips and improved chemistry, the pricing has become comparable to the majority of other clinical diagnostic procedures. There exists a number of different NGS platforms. Each has its particular virtues but from a user perspective, it is meaningful to distinguish between short- [7] and long-read sequencing [13]. Short-read protocols generate reads of < 300 base pairs (bp), whereas long-read sequencing can provide uninterrupted reads ranging from 10 kbp to several megabases depending on the technology [13]. Long-read sequencing improves the sequence phasing and it is the preferred method for solving larger haplotypes and detection of complex structural variants and repeats. In comparison short-read sequencing is the most widely applied method for detection of smaller variations because it is fast and provides high -accuracy and -sequencing depth for smaller, as well as, larger variants [14] at a low cost per base. Short reads can also be employed for applications aimed at counting the abundance of specific reads and expression analysis. Whereas, short read instruments are far more common, both platforms are appreciated and, in many laboratories, they supplement each other. Procedures are being developed that will facilitate the generation of long reads on short-read instruments, underscoring the complementarity of the methods. Nowadays short-read WGS protocols routinely provide 10 times (10X) coverage of more than 95% of the human genome and a median coverage of 30X in a single analysis, and this is generally considered sufficient for germline analysis. In order to identify minority clones, tumour analysis requires about 90X coverage. WGS is normally performed as paired-end sequencing, which enables more accurate read alignment and detection of structural rearrangements. Current, WGS protocols take approximately four working days and they are less labour-intensive than panel or exome sequencing due to the absence of the capture and amplification step.

Due to the impressive technical performance of the many commercial solutions and the defined laboratory procedures, clinical WGS workflows can be accredited according to ISO 15189. Great efforts are made to automate procedures, since sample exchange is a significant source of error. Because WGS is unlikely to be repeated, and may be reanalysed if new clinical insights or causes of a particular disease are discovered, it is crucial to reduce the risk of sample exchange. The frequency of sample exchange is incompletely documented, but based on our experience from panel sequencing, we estimate that it occurs in approximately 1 out of every 3000 samples. To mitigate the risk of sample exchange, we recommend that single nucleotide polymorphism (SNP_ID) surveillance is included for all WGS samples. This means that an independent patient sample undergoes panel analysis of a small number of highly polymorphic SNPs in parallel with the WGS sample, and that WGS data are only released for interpretation if the IDs match, and only match, the same individual. Additionally, manual pipetting steps may be video monitored to enable the tracking of sample mixing. These measures have not only improved the detection of sample exchanges in the laboratory, but also prior to arrival at the facility. Moreover, they provide an additional check for the correct family identification of trio samples.

## Bioinformatics

WGS requires a robust computational infrastructure to ensure fast and reliable data processing [15]. While the turn-around-time for patients with stable conditions may not be critical, neonates or patients in unstable and severe conditions may require prompt analysis. Also, tumour analysis should also be swift in order to begin treatment as soon as possible [16]. Consequently, clinical WGS pipelines must fulfil a set of requirements concerning both the physical computational and the software application infrastructure. The challenge is illustrated by the amount of data produced by WGS compared to large gene panels or exomes. Whereas, panel and exome analyses generate about 0.15GB and 5GB raw data, the output of a WGS analysis is about 30GB. The corresponding variant files (.vcf) from gene panels or exomes are about 7E-05GB and 0.04GB, whereas, WGS come near 1GB which corresponds to an increase in data of 13.000- and 24-fold, respectively.

Figure 1 depicts the three most important steps in the data analysis pipeline: 1. mapping, 2. calling and 3. Interpretation. Interpretation, is in principle independent of the variant calling and is performed by dedicated staff using third-party software with a graphical interface that enables interactive and flexible sorting annotation and filtering of the data. The creation of standardised end-to-end variant calling workflows was pioneered by the open-source Genome Analysis Tool Kit (GATK) [17], which forms the basis for many clinical, academic, and national WGS centres. However, a number of commercial hardware-accelerated solutions such as DRAGEN™ and Sentieon® [18], as well as prediction-based approaches are also available [19, 20]. None of these solutions are plug-and-play, and centres performing large-scale WGS analysis should be prepared to participate in pipeline development and maintenance to provide a safe, reliable and updated analytic environment.

In a production environment considerable engineering effort is dedicated to data handling, such as book-keeping of IDs and linking clinical metadata. From these, at times complex, sources of information it is possible to automate a specific pipeline run, and transfer a tailored set of output files to their proper destination. The data management includes renaming files, generating delivery notifications, logs, archives and clean-up of hundreds of intermediary files. In a clinical environment the system integration needed for the correct information flow often crosses multiple firewalls, domains and databases, and daily operation depends on support from a clinical production grade IT-organisation. Pipeline managers like *snakemake* [21] or *nextflow* [22] are important to orchestrate jobs and processes in the pipelines which may consist of several hundred steps - each with distinct resource requirements and parallelisation potential. In this environment commercial hardware-accelerated solutions that runs each sample serially can sometimes experience problems and tools that can run in parallel based on generic computers may be faster for the last finished sample on a high-performance computer cluster (HPC). More recent sequencing machines with build-in data processing hardware and closed end-to-end workflows may also bring limitations on how to reprocess samples and integrate historic data to advance diagnostics. Since the bottleneck in processing and variant calling from short-read sequencing often is the data-transfer times it is worthwhile to consider the design of the data storage system and the connection to the compute units, as well as cost-efficient storage tiers for active and archived data, respectively. Cloud solutions can be difficult to engineer for fast WGS, because the data is physically generated, and sometimes also physically stored, far from the computation units. Taken together, the initial and very general tasks of demultiplexing pooling barcodes, read alignment and marking of duplicate reads can be performed close to - or inside - the sequencing machine and will result in considerably less data transfer needs, but for more specialised tasks that are impacted by local optimisation and historic background data an HPC or cloud solution is needed.

Test, validation and accreditation is equally critical for bioinformatics production as it is for laboratory. For germline variant calling, initiatives like the Genome in a Bottle project have made it possible to benchmark and optimize tools, and there are even competitions from the American Food and Drug Administration (“FDA challenges”) in place to encourage such optimization. However, there is still no established reference for somatic variant calling. While the 1+ Million Genomes initiative [23] and the Somatic Mutation Working Group of the Sequencing Quality Control Phase II Consortium [24] have begun to address this building a community standard truth set of somatic variants remains a challenging task. Instead, in-house data comprising hundreds of manually curated somatic mutations must be reanalysed each time a new modality is implemented. A similar need of standard exists for detection of copy number alterations and inversions, and it is still a major challenge to call these in bioinformatic pipelines. Current tools are unable to detect all CNVs [25, 26], and because each algorithm has a specific recall bias so the only viable solution is to combine tools with different strategies. Since the output contains thousands of called variants, most of which could be correct but are not clinically relevant, it is also necessary to employ a large background panel from uniformly processed historic in-house samples to remove irrelevant calls. Correspondingly, somatic variant callers like Mutect2 (https://gatk.broadinstitute.org/hc/en-us/articles/360037593851-Mutect2) and GATK-gCNV [27] also rely on pre-processed background cohorts in a panel of normals, and it is recommended to avoid using public data because it may have a different noise modality. Most clinical bioinformatic units therefore relies on the access to a large harmonized in-house database of historic patient data. As described below polygenic scores and somatic mutations signatures are also expected to become part of the WGS pipelines. Regardless of the computational method the calculations are highly dependent on the sequencing platform, library preparation, sequencing depth and variant calling and filtering pipeline. Consequently, computations of mutational signatures [28] and polygenic scores [29] (see below) should be interpreted with great caution - and always - in relation to a scale of historic cases potentially blinded as quartiles if per-sample information cannot be displayed. In the very last step of the bioinformatics pipeline, it should also be recalled that most interpretation softwares do not require filtering before uploading and e.g., filtering on genome frequencies [30] should only be applied in the analysis software by the clinical interpreter as a conscious decision. Finally, as always - it is important to underscore that clinical data are sensitive and data privacy and safety should be highly prioritized in the WGS bioinformatics solutions.

## Data filtering and interpretation

**Fig. 2:**
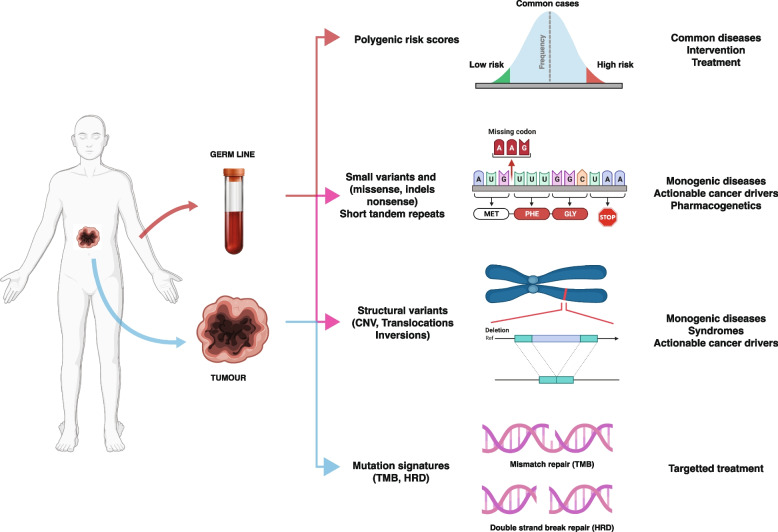
Clinical applications of WGS. Whole Genome Sequencing (WGS) finds its primary clinical applications in diagnosing rare diseases and pinpointing actionable somatic variants within tumors. Beyond these crucial roles, WGS serves to unveil polygenic risk scores (PRS) and pharmacogenetic profiles. The spectrum of rare diseases and somatic variants encompasses both small and structural variations, all discernible through WGS data analysis. WGS also enables the identification of trinucleotide repeat expansions prevalent in neuro-muscular and degenerative diseases. Additionally, it sheds light on polygenic and pharmacogenomic profiles, elucidated by the presence of widespread small common variants. In a comprehensive approach, WGS not only captures the intricate details of genetic makeup but also unveils tumor signatures by deciphering distinctive patterns within somatic variants. Human insert was created with BioRender.com

Whole-genome sequencing (WGS) is widely employed to diagnose rare [31–35] and undiagnosed diseases [36] and identify actionable cancer drivers and signatures. The different clinical applications and the type of analyses that are implicated in the diagnostics are shown in Fig. 2. There are several reasons why whole-genome sequencing (WGS) is becoming the preferred method for genetic analysis over alternative methods such as panel and exome sequencing. Firstly, WGS detects more variants not only in the large noncoding parts of the genome but also in exons due to a superior mapping quality [31, 36, 37]. Secondly, WGS captures copy number variations and structural rearrangements as well as mutation signatures and polygenic scores. Finally, WGS can be considered a lifelong investment that may be revisited for different clinical purposes and reanalysed when novel pathogenic variants and disease-causing genes emerge [38]. It has been estimated that about 250 new disease genes are discovered every year, and that up to 6000 Mendelian conditions remain to be discovered [39, 40]. As a direct illustration of the situation, it is worth mentioning that almost half of the variants identified in the recent UK and Ireland rare pediatric disease WGS study [37], where unknown by the time the study was initiated. The relation between human genetic variation and disease is summarised in Text Box 1.

**Text Box 1:** Human genetic variation and disease

The human genome is composed of 3.2 billion base pairs of DNA, organized into 23 pairs of chromosomes [2–4]. In addition to the nuclear genome there is also a small amount of maternal DNA located in the mitochondria. Only about 1,5% of the genome sequences consists of protein coding exons [41]. The remaining 98% of the genome is made up of non-coding regions, which include regulatory elements, repetitive DNA sequences, and other functional elements [42]. While there is general consensus that we have about 20,000 protein-coding genes, the size of the proteome is still debated [41, 43]. Moreover, a numbers of non-coding RNAs such as micro RNAs and long non coding transcripts are also produced but their number and biological significance are with few exceptions uncertain [44]. Like any other species humans are under constant selection and genetic variation is an integral part of the evolution. We continuously acquire both positive adaptive germ cell mutations as well as neutral and disease causing variants [45]. Mutations result from radiation, environmental stress factors and deficient DNA repair [46] and they locate to all parts of the genome [47] albeit with varying frequency. On average a human genome accumulate about 75 mutations per generation [45]. Dominantly inherited variations leading to lactase persistence has for example allowed adult northern Europeans to digest milk [48] and the caspase-12 gene is polymorphic for a stop codon, that makes carriers more resistant to severe sepsis. We can also observe how the Black Death shaped genetic diversity around particular immune loci such as *ERAP2* and *CTLA4,* highlighting how natural selection may have played a role in present-day susceptibility towards chronic inflammatory and autoimmune disease [49]. Finally, it is clear that genes encoding transcription factors and RNA binding proteins which are essential for fetal development are subject to a strong selective pressure as illustrated by their low or entirely absent occurrence of loss of function variants [30]. In genetic terms humans are 99.9% identical to each other. The remaining 0.1% of our genome corresponding to ~ 3.000.000 simple variants distinguish us from another. Among these ~ 45.000 (1.5%) are found in protein coding exons [2]. In addition, numerous structural variations, such as copy number variations (CNVs) and structural variations (SVs) may contribute to our genetic diversity [50, 51]. From a medical perspective, this genetic variation significantly influences individual susceptibility and disease development. The impact extends to pharmaceutical side effects and clinical outcomes, underscoring the integral role of genome sequencing in personalized medicine. Both rare (< 1% minor allele frequency) and common variants (> 1% minor allele frequency) contribute to the risk of developing a disease, and they can sometimes interact with each other in complex ways. From a diagnostic point of view this is one of the major challenges for the current interpretation of WGS data. Common variants are typically associated with a small increase in disease risk, but because they are so common, they can have a significant impact on the population as a whole. At the individual level the presence of numerous common variants may generate a significant risk for a particular disease and their cumulative effect is captured by the current polygenic risk scores (PRS). Rare disease associated variants, on the other hand, with few exceptions occur at a much lower frequency in the population, often far less than 1% of individuals. Most of the rare variants that are considered in diagnostics locates to the coding exons and alters or reduce the function of the encoded proteins. In families they exhibit a mendelian segregation pattern in the families, but they may also occur as de novo variants. During the past decade genome-wide association studies (GWAS) have associated thousands of common-variants to various diseases and traits, and in the same a series of large-scale sequencing studies have recently started to identify rare-variant associations [52–54]. A surprising finding has been that for a particular trait, common and rare variants appear to be mechanistically convergent [55]. The relative contribution of rare variants to the total genetic burden may be relatively small but rare variants may serve to improve the fundamental understanding of the disease pathogenesis and define possible targets of treatment.

### Rare monogenic disorders

**Fig. 3:**
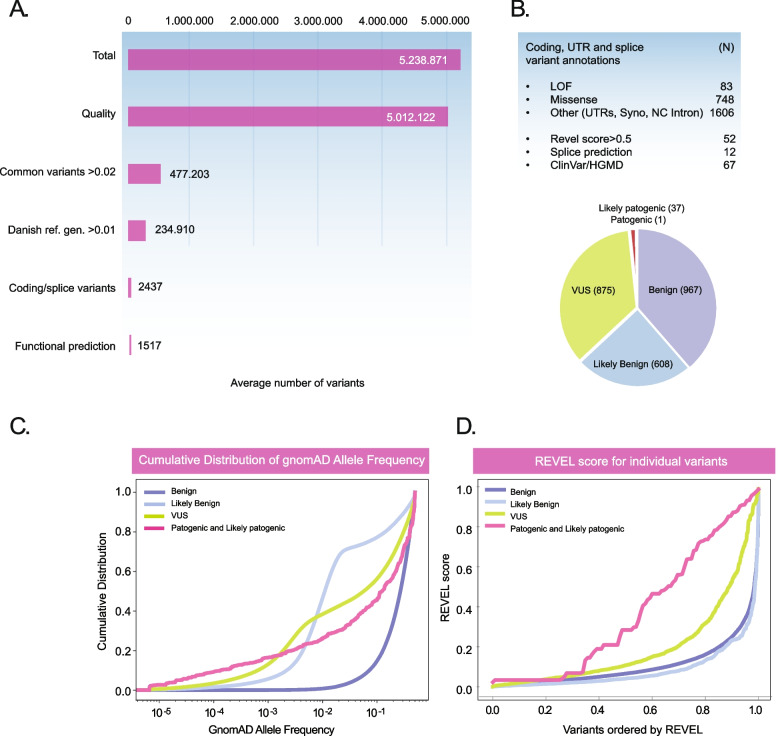
Variant analysis of patients with rare diseases. Panel **A** Overview of the filtering steps and the number of variants in rare disease patients referred for WGS analysis (means of 6 patients). The total number of variants in each patient is just above 5 MIO. The analysis begins by elimination of ~ 200.000 low quality variants. Subsequently, common variants with an allele frequency above 2% are excluded, since these are considered unlikely to explain the occurrence of a rare disease. Known pathogenic variants are retained. Since gnomAD may not represent all common variants, variants are moreover filtered against a local (Danish) reference genome and this further reduces the number of variants to about 200.000. Thereafter, the analysis is focused on coding and splice site variants and on average this reduces the number of variants to ~ 2400. Application of additional filters e.g., omitting ACMG/AMP benign variants or those with low REVEL scores further brings the number of variants down to ~ 1500. Panel **B** On average the patients exhibit 83 loss of function (LOF) variants and 748 missense variants. The remaining variants belonged to other categories such as variants in the UTRs and deep into the intron. Finally, on average 67 variants were previously registered in ClinVar or HGMD and information on these can be readily retrieved and used in the interpretation. The pie chart below shows the ACMG/AMP classification of the variants showing that only a minority are classified as pathogenic and likely pathogenic (< 2.5%). On average only a single pathogenic variant is identified. In many cases the variant represents a recessive heterozygote variant with no obvious relevance for the patient’s disease. Almost one third of the variants represents variants of unknown significance (VUS). Panels **C** and **D** shows the total cumulative distribution of gnomad allele frequencies and REVEL scores of ACMG/AMP scored variants (from Varseq) among 63 unrelated patients, respectively. Intergenic variants were filtered away and any variant which had conflicting classifications was removed. Moreover, variants with an allele frequency of more than 0.5 or for which an allele frequency could not be found was removed. The results illustrate that allele frequency is relatively effective in excluding benign variants, whereas likely benign and VUS are not effectively separated from the likely pathogenic and pathogenic variants by frequency filtering. The REVEL score combining ﻿pathogenicity predictions from 18 individual scores, in contrast, is clearly discriminative and high scores are enriched among pathogenic variants. About 25% of the VUS exhibit REVEL score above 0.5 that may warrant further analysis of these variants. The number and details of variants in the plots is summarized the attached Supplemental data

The output of a single WGS is about 5 million variants and the data interpretation begins by importing variants (.vcf files) into one of the many commercially available or in-house designed software tools that makes it possible to filter and annotate the variants. Filtrations, include exclusion of variants based on their quality, population frequency, functional impact and clinical relevance, in order to focus on variants with a putative causal role for the patient’s disease. A number of analytical approaches and filtering schemes have been put forward by various expert groups and initiatives and these may serve as a fine starting points for the interpretation units [56–60]. Figure 3 provides an example of a filtering scheme and how it affects the selection of variants.

In principle the analytic strategy may be genotype-driven or symptom/disease (phenotype)-driven [56]. Genotype-driven analyses are focused on the identification of pathogenic variants loss of function variants, whereas the symptom/disease driven analyses focus on variants that are compatible with the inheritance pattern. There is no strong delineation between the two approaches and they are often combined. In cases where the diseases have a well-defined symptomatology an *in-silico* gene panel of known disease related genes can moreover be applied at an early stage to focus the analysis even further. In this way the exact analytical approach depends on the clinical presentation and whether the patient represents an isolated case or has a familial predisposition.

For children with healthy parents, a trio examination can be performed to identify pathogenic de novo heterozygous or compound heterozygous variants that are compatible with the clinical diagnosis [61]. The diagnostic success is higher for trios than singletons and usually only 10–30 variants have to be scrutinized [37]. In cases with a familial predisposition, relevant affected and healthy family members can be included to subtract variants from healthy subjects and focus on shared variants in the probands and affected family members. Analysis of singletons is the most variable and challenging. Approximately 90% of variants are common variants with a frequency greater than 2% and these are typically filtered out. Known pathogenic variants should obviously be retained for downstream analyses (Fig. 3C). The remaining ~ 500,000 variants may be further filtered based on minor allele frequency and their location and significance focusing on nonsense, indels, proximal splice-site, and missense variants with a frequency below 1% or 2%. This normally reduces the number of variants to around 2500 or fewer, especially, if combined with relevant gene panels (Fig. 3A). About 40–80 variants normally represents pathogenic loss of function variants (LOF) that may be assessed directly (Fig. 3B). Factors such as ethnicity or founder effects occasionally warrant changes to the general filtering scheme and it is important to note that the expected frequency of a pathogenic variant in the population depends on the penetrance of the variant or gene.

The ACMG/AMP classification criteria [59, 62] are widely used for prioritizing variants based on their pathogenic significance. Based on characteristics such as allele frequency, case data, functional data, and data sources, variants are categorized into five classes: 1. benign, 2. likely benign, 3. variant of uncertain significance (VUS), 4. likely pathogenic, and 5. pathogenic. The prioritization of VUS and putative pathogenic variants involves several considerations. As shown in Fig. 3C, allele frequencies are not very discriminative between VUS and benign variants and a number of other features needs to be considered in order to classify VUS. It is obviously important if the variant has been observed in other patients and whether there is direct evidence linking the variant to the patient’s disease or symptoms. This information can sometimes be obtained from databases such as The Human Gene Mutation Database (HGMD) or ClinVar (Table 1), or from the scientific literature. Moreover, the presence of homozygous individuals in population databases such as The Genome Aggregation Database (*gnomAD*) may support that the variant is benign. Additionally, search engines like PubMed, OMIM, and Find Zebra are also useful in establishing the significance of a variant or gene. Many commercial software tools even offer access to knowledge databases, providing more systematic reviews of the literature and databases that allow the interpreter to narrow down genes and variants associated with particular diseases or symptoms. Finally, predictive functional scores such as the REVEL score [63] (Fig. 3D) and the recent AlphaMissense prediction tool [64] (see below) are likely to play a larger role in the future. Note that the available database solutions are not standardized or accredited, and it is important that the interpreter document the reasons for the classification of a particular variant. If the analysis fails to identify an association between a gene and a disease, the molecular pathway in which the protein functions may eventually be considered. Pathway analysis is still in its early stages, and associations should be confirmed by functional analysis to support that a variant is in fact pathogenic. Finally, it is important to mention that VUS and even clear loss of function variants sometimes are located in genes of unknow significance (GUS). GUS are defined as genes without validated association with a given phenotype [59] and as a result of the uncertainty current guidelines recommend that any variant in GUS is reported as VUS. Rare, predicted damaging variants in GUS are obviously of great interest because they may eventually lead to the discovery of new disease gene. It is important that they are reported to relevant databases such as the Matchmaker Exchange that promote Genomic discovery through the exchange of phenotypic & genotypic profiles [65] (www.matchmakerexchange.org) or even for improved functional annotation in MaveDB [66].

**Table 1:** Biomedical databases relevant for clinical WGS

Database/Resource	Web address	Content
ClinGen	https://clinicalgenome.org/	Clinical relevance of genes and variants
ClinVar	www.ncbi.nlm.nih.gov/clinvar/intro/	Database of genomic variants with public submissions of variant interpretations and disease relations.
Cosmic	https://cancer.sanger.ac.uk/cosmic	Catalogue Of Somatic Mutations In Cancer
dbSNP	https://www.ncbi.nlm.nih.gov/snp/	Contains human single nucleotide variations, microsatellites, and small-scale insertions and deletions
Ensembl	https://www.ensembl.org/index.html	Genome browser for vertebrate genomes that supports research in comparative genomics, evolution, sequence variation and transcriptional regulation
Find Zebra	https://www.findzebra.com/	Tool for helping diagnosis of rare diseases. It uses freely available high quality curated information on rare diseases
Genomics England	https://www.genomicsengland.co.uk/	Comprehensive site describing the progress of the UK sequencing initiative. Site contains usefull overviews over gene panels and diseases.
Geo		Repository supporting MIAME-compliant data submissions. Array- and sequence-based data
gnomAD	https://gnomad.broadinstitute.org	Exome and genome sequencing data with allele frequencies from a wide variety of large-scale sequencing projects
GTEX	https://gtexportal.org/home/	Comprehensive public resource to study tissue-specific gene expression and regulation. Samples were collected from 54 non-diseased tissue sites across nearly 1000 individuals, primarily for molecular assays including WGS, WES, and RNA-Seq.
HGMD	https://www.hgmd.cf.ac.uk/ac/index.php	Collate all known (published) gene lesions responsible for human inherited disease
Human Phenotype Ontology (HPO)	https://hpo.jax.org/app/	Provides a standardized vocabulary of phenotypic abnormalities encountered in human disease
Matchmaker Exchange	https://www.matchmakerexchange.org	Genomic discovery through the exchange of phenotypic & genotypic profiles
MaveDB	https://www.mavedb.org/	Collection, distribution, and analysis of variant effect maps
MedGen	https://www.ncbi.nlm.nih.gov/medgen/	Organizes information related to human medical genetics, such as attributes of conditions with a genetic contribution
NCBI	https://www.ncbi.nlm.nih.gov/	The National Center for Biotechnology Information advances science and health by providing access to biomedical and genomic information
OMIM	https://www.omim.org/	Compendium of human genes and genetic phenotypes
RefSeq	https://www.ncbi.nlm.nih.gov/refseq/	A comprehensive, integrated, non-redundant, well-annotated set of reference sequences including genomic, transcript, and protein.
The Cancer Genome Atlas Program (TCGA)	https://www.cancer.gov/ccg/research/genome-sequencing/tcga	The Cancer Genome Atlas (TCGA) has molecularly characterized over 20,000 primary cancer and matched normal samples spanning 33 cancer types.
UCSC genome browser	https://genome.ucsc.edu	Interactively visualize genomic data
Uniprot	https://www.uniprot.org/	Comprehensive and freely accessible resource of protein sequence and functional information.

**Fig. 4:**
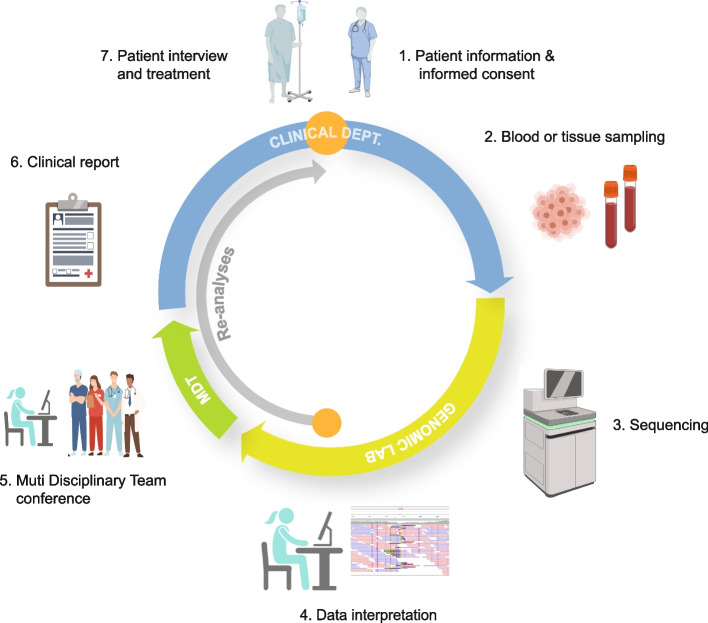
WGS from patient to clinical report. The journey of Whole Genome Sequencing (WGS) commences and concludes at the patient’s bedside. Upon the attending physician’s assessment, a WGS analysis is deemed potentially beneficial for offering crucial clinical insights, either through diagnosis or by presenting alternative treatment options. Following comprehensive patient briefing and obtaining consent, a sample of whole blood or tumor is dispatched to the specialized laboratory equipped for WGS. Within the genomic laboratory, the sequence data undergo meticulous analysis by the skilled staff. Putative disease-associated variants are subsequently deliberated with the attending physician and, if necessary, a multidisciplinary team comprising medical professionals from pertinent specialties, forming a Multidisciplinary Team (MDT). Specialties include pathology, clinical genetics, immunology, and more. This collaboration aims to establish a conclusive diagnosis and assess the clinical relevance of identified variants. The conclusive clinical report is then transmitted to the clinical department, where the attending physician shares the results with the patient. This communication includes a comprehensive discussion of the implications for the patient and their condition, along with recommended actions. In instances where the initial analysis fails to pinpoint disease-causing variants, the stored WGS data undergoes periodic re-analysis (inner grey arrow). This ongoing process ensures the continuous integration of new knowledge, potentially leading to a diagnosis without the need for additional hospitalization and sampling. Furthermore, throughout the treatment course, various clinically relevant information, such as pharmacogenetics, may be extracted to enhance the overall patient care experience. Inserts were created with BioRender.com

From a clinical standpoint, VUS obviously represent a dilemma because their causative role in a particular disease is not fully established. Some argue that VUS should simply be eliminated from the analysis [67, 68] and await further evaluation, while others emphasize the risk of leaving patients without a diagnosis if a clinically relevant VUS is disregarded. The number of VUS will likely decrease over time when databases accumulate more data and our understanding of disease pathogenesis improves. Currently, there are no definitive guidelines for all clinical situations, so common sense and clinical experience are important. In many WGS centers, variants are discussed with the attending physician or in multidisciplinary teams to ascertain their clinical relevance. In general, only pathogenic (class 5) and likely pathogenic (class 4) variants are included in the final clinical report. Finally, it is convenient to include details about the sequencing and analysis method used and the composition of in silico gene panels in the report for future reference. Figure 4 illustrates the general scheme of clinical WGS reporting.

### Somatic variant analysis

WGS of tumour and germline DNA in combination with RNA sequencing-based expression analysis is widely used to identify actionable tumour drivers and host factors. WGS is the preferred method for tailored treatment because it potentially uncovers both the small somatic tumor variants, CNVs and facilitate the detection of characteristic mutation signatures such as HRD and TMB. The complete map of somatic mutations and alterations in gene expression patterns provides integrated information for selection of the optimal treatment.

Somatic variant calling requires a whole blood sample for germline variants and a tumour sample for somatic variants and transcriptome analysis. Somatic variants are identified by subtracting germline variants from the tumour sequence. It is not recommended to exchange the blood sample for a panel-of-normals germ-line variant set because of the higher noise level. Typically, tumours exhibit about 500.000 somatic variants and as described for the germ line analysis the variants undergo a series of filtering’s based on their frequency, call quality and read depth as well as their cancer relevance before interpretation. After filtration between 20 and 1500 variants are normally eligible for further evaluation. Based on their significance in cancer, prognosis, and/or therapeutics somatic variants may be classified into four tiers. Tier I, represents variants with strong clinical significance, Tier II variants with potential clinical significance and Tier III variants of unknown clinical significance whereas Tier IV is benign or likely benign variants [60]. Actionable somatic variants are subsequently be queried in relevant databases [69, 70]. Many laboratories also report the tumour mutation burden (TMB) score that is associated with immune cell infiltration and increased sensitivity to programmed cell death-1 (PD-1) or PD-1 ligand (PD-L1) blockade. Finally, a homologous recombination deficiency (HRD) signature linked to poly(ADP ribose) polymerase (PARP) inhibitor sensitivity [71–73] may also be generated from the WGS data.

### Polygenic risk scores

Genome-wide association studies have revealed that common disorders such as type 2 diabetes, cardiovascular diseases, and some cancers, are associated with combinations of common variants each providing a small increase in risk for the particular disease [74–78]. The polygenic risk burden is combined into a polygenic risk score (PRS) that can support diagnosis, screening, and intervention at early stages of disease. The number of variants included in the PRS can range from a few (< 10) to thousands of variants, and while the discriminative ability of PRS in the general population has been debated, larger and more diverse studies, as well as refined computational strategies, have revitalized the clinical interest in PRS [29, 76–78]. Cheap chip-based assays are useful for PRS analyses, but WGS may become an appealing alternative because it will identify both common and rare variants that potentially may contribute to the genetic makeup of a diseases. Extraction of data for individual PRS can be integrated into the WGS pipeline and added automatically to the clinical report, providing a comprehensive genetic profile of the patient.

##  In-silico prediction and functional testing of variants

**Fig. 5:**
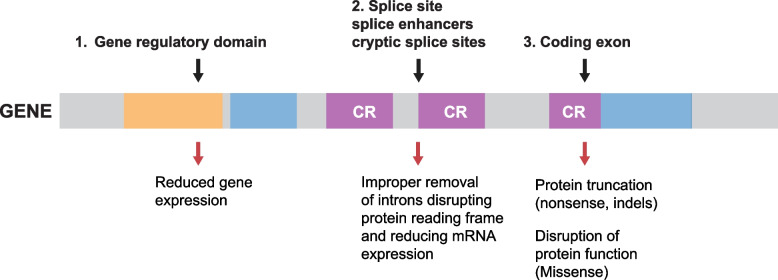
Genomic localization of variants and their functional consequence. 1. Germ-line variants located in the gene regulatory domains such as promoters or locus control regions will affect the level of gene transcription. In most instances variants in the promoters disrupt the binding of trans-acting factors thereby reducing expression of the gene. The composition of regulatory motifs is in many instances incompletely understood and it is in general difficult to predict the consequence of these variants. A few diseases exhibit unstable trinucleotide repeat sequences in the promoter, that when expanded is known to impair transcription. The functional significance of promoter variants is normally demonstrated by loss of expression (LOE) via RNA sequencing or measurement of the encoded protein. Repeat expansions may also be directly discerned from the WGS data 2. Variants located at the canonical splice donor (GT) or acceptor (AG) sites or at a known A – branch-site are in general pathogenic since these strongly conserved sequences are essential for splicing. Variants located deeper in the intron or in the connecting exons can also disrupt splicing due to disruption of enhancer or silencer motifs but the significance of these variants is more difficult to predict. The evaluation of these variants in general requires minigene analysis and/or RNA sequencing. 3. Coding nonsense or frameshift variants lead to premature translation termination and shortening of the encoded protein. In most cases this can lead to loss of function (LOF). Missense variants and small indels may disrupt protein function in a number of different ways such as reducing enzymatic activity, stability, localization or structure and macromolecular assembly. Consequently, the evaluation of these variants requires deep insight into the proteins function and in many instances various kinds of functional analysis is necessary in order to classify the variants as pathogenic. Since the functional significance of a particular variant may be difficult to predict - even for canonical splice mutations and LOF variants - it recommended that all classes of variants undergo evaluation according to ACMG/AMP criteria in order to determine pathogenicity

With the increasing diagnostic sequencing and identification of new disease genes, the number of VUS that needs to be considered will increase [68]. Consequently, there is great focus on *in-silico* and in vivo analyses to better understand the significance of these variants. Figure 5 provides a schematic representation of the functional consequence of various types of mutations.

### Predictive scores and protein structure

Missense variants are commonly assessed based on their frequency, conservation, and the location and type of amino acid substitution. Predictive scores that take this information into account are being developed, and among the most widely used are Polyphen [79], SIFT [80], and CADD [81]. The REVEL score, in particular, combines scores from a wide range of tools and provides a relatively high enrichment of pathogenic variants [63]. Precomputed REVEL scores are available for all possible human missense variants and can be integrated into the clinical analyses. With the rapid accumulation of AI-driven protein structures in the AlphaFold Protein Structure Database [82], many hoped that structural predictions could be used for assessment of Variants of Uncertain Significance (VUS). Although, initial attempts were not entirely successful [83, 84], the recent AlphaMissense (AM) algorithm, represents a major leap forward [64]. AM integrates information of evolutionary conservation and protein structure - both of which are intimately linked to protein function - and classifies variants as likely -pathogenic or -benign. The precision of AM is thus far unmatched and the algoritm holds great potential to facilitate the classification of VUS.

### mRNA expression and splicing

The processing of primary RNA transcripts from transcription to translation and decay involves a series of well-characterized steps that can be affected by both coding and intronic variants (Fig. 5). In addition to the canonical GT-AG donor and acceptor sites, variants may involve exonic splice enhancers and/or intronic silencers or generate novel splice slice sites. The percentage of variants that affect pre-mRNA splicing varies among diseases ranging from 10 to 50% (reviewed in [85]) and studies have indicated that as many as 25% of exonic mutations may have an effect on splicing [86, 87]. RNA sequencing reveals the expression of individual alleles and the exonic composition of the transcripts and may uncover that coding variants are located in exons failing to be expressed in the relevant tissue. Calling of fusion genes from RNA-seq data is also important. In particular for cancer diagnostics, because the fusion protein may be targeted by drugs. Given the relatively poor accuracy of fusion gene calling [88] it is recommended to use a number of fusion calling tools and rely on a weighted consensus score to prioritise the predictions. For selected clinically relevant fusions a whitelist may even be incorporated in the consensus calling so low frequency targetable fusions are not overlooked. Finally, minigene analysis remains a paradigm for the functional categorization of splice variations [89]. Several in silico prediction tools have also been developed to predict whether a particular variant is likely to affect splicing [90–92]. In silico prediction cannot stand alone but should prompt further analysis of RNA sequences or minigene splicing.

### Protein function

The classification of a coding VUS should ultimately rely on the characterization of the protein’s function. Although, functional testing of an enzyme may be relatively straightforward, complex processes such as homologous recombination requires the assembly and concerted effort of several factors. As a result, there is no one-size-fits-all approach to functional testing and the analysis varies from disease to disease and from protein to protein. Variants implicated in metabolic diseases may e.g., be directly visualized by NMR, whereas disruption of protein assemblies can be examined through conventional pull-down experiments. Dislocations may be visualized through the expression of the factors in suitable cell systems followed by microscopy. Some cell systems, such as induced pluripotent stem cells (iPSc), may even reconcile tissue-specific effects [93]. Many of the assays are difficult to perform in a routine clinical context, and to solve this problem more systematic screenings of variants are emerging. A recent example of this is the CRISPR-based saturation genome editing screening and classification of over 4000 BRCA1 variants [94, 95], which has facilitated diagnostics of woman with breast ovarian cancer significantly.

## Results from the clinical application of WGS

For rare diseases pediatric- and clinical genetics departments are major requestors, but in principle any medical specialty, may encounter patients with diseases where conventional workup has failed to provide a diagnosis. Large series of patients with rare diseases [31, 32, 35, 36, 96] have demonstrated an average diagnostic yield of ~ 25% for probands. Somewhat over 10% of these diagnoses were caused by variants in genomic regions that would not have been identified by other methods. Moreover, a few percent involved coding variants in regions of low coverage on exome sequencing [31]. The results are in line with data from screening of undiagnosed patients, where about half of the patients who receive a diagnosis from WGS have previously undergone exome sequencing [36]. The diagnostic yield varies across different patient groups, ranging from a few percent for respiratory and some hematological disorders to 40–50% for hearing and ophthalmologic disorders, intellectual, and neurodevelopmental disorders. For patients with heart disease or immune deficiency, the diagnostic yield is 20–30% [31]. In a recent study of rare paediatric disorders - a diagnosis was made in about 40% of the probands of whom 76% exhibited a pathogenic de novo variant [37]. The diagnostic yield is highest among probands analysed in trios and for patients with more pronounced symptoms. On average 2.5 and 1 candidate variant were reported in singletons and probands analysed as part of trios, respectively. Children with intellectual disability, neurodevelopmental disorders, and complex syndromes usually require a complex diagnostic workup, and since the WGS results are positive front-loading of the analysis during the diagnostic work-up have been recommended [97–99]. Another important experience from the use of WGS is moreover that the analysis may uncover unique presentations of known diseases or a completely new disease. In this way WGS may have a significant influence on future disease classification and identification of novel syndromes.

For oncological patient’s comprehensive tumour characterization has demonstrated the effectiveness of tumour sequencing in conjunction with transcriptome analysis to support targeted treatment. WGS uncovers actionable tumour variants in approximately two thirds of metastatic tumors but it should be underscored that there is large variation among tumor types [69, 100–103]. In addition, germ line sequencing has revealed that a significant number of cancer patients carry predisposing mutations in tumour-suppressor genes [104–106]. The combination of tumour and germ line sequencing has significant potential for improving patient outcomes in cancer treatment, although, there is a strong need to improve the prioritization and characterization of variants in order to increase the response rate of the new drugs.

## Ethical concerns

Like any other medical tests, genome sequencing, raises ethical dilemmas for the society and patients. A number of the concerns such as privacy and confidentiality issues, consent, patients psychological stress, involvement of biologic relatives, social stigmatization, insurance and employment issues are shared with genetic testing in general [107–110]. Genome sequencing, however, also presents a few unique challenges due to the vast amount of information generated. We may not be in a position, where we can fully understand the implications of the data and there is moreover greater potential for incidental findings. This demonstrates the necessity of in-depth information to the patient prior to the analysis (Fig. 4). Moreover, the permanent and complete nature of the data makes it difficult to foresee future applications and dilemmas for the patients [111, 112] (CADTH report). Finally, privacy concerns and data-sharing issues are more challenging because data management often involves third parties outside the health-care systems. It is important that health-care providers take responsibility for safe data storage and prevention of unauthorized use of patient data. Since WGS technology is relatively new and is relevant in many medical specialties, we also want to highlight the importance of proper guidelines and education of the staff in general. MDs and nurses close to the patients should be comfortable with the technology in order to inform the patients.

## The way forward

After the initial discovery and great expectations there is often a period of debate before the benefits of new technologies become evident. It is sometimes argued that WGS produce too much data that we are unable to interpret. In some way this is correct, but in our opinion, it should be regarded as an opportunity rather than a problem and prompt us to increase our efforts to understand disease pathology and genetics even deeper. One of the most important objectives for the fields is to improve variant interpretation and annotation. This will require integration of clinical data, functional studies, population databases, and extensive data sharing and development of computational tools. WGS data should moreover be further integrated with transcriptomics, epigenomics, and proteomics, in order to provide a more comprehensive understanding of disease mechanisms in Text box 1. By combining multiple layers of genomic information clinicians will be able to identify functional variants, regulatory elements, and pathways associated with diseases, enabling more accurate diagnoses and targeted treatments. Compared to a number of conventional methods WGS has also been considered expensive and to require huge storage capacity. The need for storage and high-performance computing is a concern but should perhaps be perceived in a broader context and regarded as an investment in precision medicine. Moreover, the high-performance computing infrastructure will facilitate a number of the associated research lines and stimulate the integration between clinical care and research. With respect to the clinical use of WGS, there has been a fast progress in the standards for data analysis due to the initiative of e.g., the Medical Genome Initiative [57] and ACMG/AMP as well as patient focused genome initiatives around the world. These efforts should be supported in order to advance diagnostics. Taken together, we are confident that WGS has the potential to make a difference for patients and we foresee that the clinical use will increase in the coming years.

### Supplementary Information

**Additional file 1.**

## Data Availability

According to Danish legislation, WGS files are deposited in the Danish National Genome Center (NGC) where they can be accessed after approval from NGC (https://ngc.dk/). Variant frequencies and REVEL scores are publically available from gnomAD (https://gnomad.broadinstitute.org).
