# Supplementary material for: Whole genome sequencing in clinical practice
Source: BMC Med Genomics. 2024 Jan 29;17:39. doi: 10.1186/s12920-024-01795-w (PMC10823711; doi:10.1186/s12920-024-01795-w)
Supplement: Supplementary file 1 — Additional file 1. [file 12920_2024_1795_MOESM1_ESM.docx]

**SUPPLEMENTAL DATA 1.**

Variants from 63 patients were classified using Varseq's ([www.goldenhelix.com](http://www.goldenhelix.com)) ACMG algorithm. Intergenic variants were filtered away as they could not be classified. Moreover, any variant which had conflicting classifications (e.g. Likely Benign, VUS) and variants with an allele frequency of more than 0.5 were removed. Finally, variants for which an allele frequency could not be found was removed.

The resulting dataset had the following numbers of variants in each category:

| Benign | 60893122 |
| --- | --- |
| Likely Benign | 1637535 |
| VUS/Weak Benign | 450309 |
| VUS | 26284 |
| VUS/Weak Pathogenic | 1149202 |
| Likely Pathogenic | 8016 |
| Pathogenic | 264 |
| Total | 64164732 |

VUS/Weak Benign and VUS/Weak pathogenic were merged with VUS for plots missing these distinctions. Pathogenic and likely Pathogenic were merged for plots (due to the set of pathogenic variants found being too small).

Only a subset of these variants had REVEL scores. The number of variants in each category that had REVEL scores were as follows:

| Benign | 408276 |
| --- | --- |
| Likely Benign | 12627 |
| VUS/Weak Benign | 6287 |
| VUS | 1854 |
| VUS/Weak Pathogenic | 26422 |
| Likely Pathogenic | 105 |
| Pathogenic | 17 |
| Total | 455588 |

A cumulative distribution of the allele frequencies by category was made.

A cumulative distribution of REVEL scores by category was also made.

Allele frequencies were obtained from our local annotation source including gnomAD genomes variant frequencies 3.1.2 v2 BROAD. REVEL scores were taken from our local annotation source: REVEL functional predictions.
